# Association of Wildfire Air Pollution With Clinic Visits for Psoriasis

**DOI:** 10.1001/jamanetworkopen.2022.51553

**Published:** 2023-01-13

**Authors:** Raj P. Fadadu, Marcus Green, Barbara Grimes, Nicholas P. Jewell, Divya Seth, Jason Vargo, Maria L. Wei

**Affiliations:** 1 Department of Dermatology, University of California, San Francisco; 2 Dermatology Service, San Francisco Veterans Affairs Health Care System, San Francisco, California; 3 Department of Medical Statistics, London School of Hygiene & Tropical Medicine, London, United Kingdom; 4 Department of Epidemiology and Biostatistics, University of California, San Francisco; 5 Office of Health Equity, California Department of Public Health, Richmond; 6 Now with Federal Reserve Bank of San Francisco, San Francisco, California

## Abstract

This cross-sectional study examines whether clinic visits and online search interest for psoriasis were associated with wildfire air pollution after a delayed lag period.

## Introduction

Air pollution may trigger psoriasis flares,^1^ but evidence of this association is limited. Wildfires are increasing in many parts of the world, which may contribute to worsening air pollution in some areas.^2,3^ During California wildfires in 2018 and 2020, clinic visits and online search interest in atopic dermatitis and itch increased, but online search interest in psoriasis did not.^4,5^ We investigated whether clinic visits and online search interest in psoriasis were associated with wildfire air pollution after a delayed lag period.

## Methods

As in previous work,^4^ we used 3 metrics in this cross-sectional study to characterize air pollution in San Francisco: fire status (binary indicator of whether a wildfire occurred during a week), concentration of particulate matter 2.5 μm or less in diameter (PM_2.5_) measured at ground level, and smoke plume density (range, 0-3, where 0 indicates none, 1 indicates light, 2 indicates medium, and 3 indicates heavy) based on satellite imaging. Data were collected for outpatient dermatology visits for psoriasis at an academic medical center in San Francisco from October 1, 2018, to February 10, 2019 (including the California Camp Fire), October 1, 2015, to February 10, 2016 (no fires), and October 1, 2016, to February 10, 2017 (no fires). The California Camp Fire occurred 175 miles away from San Francisco and was associated with worsened air quality in San Francisco from November 8 to 21, 2018.^4^ Statistical analysis was performed from December 20, 2021, to March 10, 2022. Outcome data on psoriasis clinic visits were stratified by age group (<18 and ≥18 years) and analyzed using generalized Poisson regression. Statistical models included 11 one-week exposure lags and were adjusted for temperature, humidity, age, year, and overall patient volume at clinics; details are included in the eAppendix in Supplement 1. In addition, we collected weekly online search interest data (ie, search value index [SVI]) from Google Trends for the term *psoriasis* in San Francisco in 2018. The study was approved by the University of California, San Francisco institutional review board, which waived the need for consent because research was deemed no more than minimal risk to participants. We followed the STROBE reporting guidelines.

Data management and statistical analyses were conducted using Stata, version 16 (StataCorp LLC) and R, version 4.0.5 (R Group for Statistical Computing). All *P* values were from 2-sided tests, and results were deemed statistically significant at *P* < .05.

## Results

We analyzed 986 clinic visits (914 visits for adults [≥18 years] and 72 visits for children [<18 years]) for 471 patients (248 male patients [52.7%]; mean [SD] age, 46.7 [19.1] years; 106 Asian [22.5%], 12 Black [2.5], and 289 White [61.1%] patients) with psoriasis (Table). The adjusted rate ratios (RRs) for adults across 11 one-week lags are shown in the Figure. For psoriasis clinic visits by fire status for adults, the earliest statistically significant increase in adult visits occurred at the 5-week lag (RR, 1.32 [95% CI, 1.02-1.70]) and peaked at the 8-week lag (RR, 1.45 [95% CI, 1.13-1.86]) and 9-week lag (RR, 1.45 [95% CI, 1.12-1.87]). No statistically significant results were found for pediatric patients. The mean weekly SVI for psoriasis from October 4, 2018, to January 30, 2019, showed an increase in search volume also starting 5 weeks after the fire and peaking at week 8 (Figure).

**Table.** Summary Characteristics of the Study Population: Visits and Patients

Characteristic	No. (%)			
	All years^a^	2015-2016 (No fires)^b^	2016-2017 (No fires)^b^	2018-2019 (Camp Fire)^b^
Total, No.	986	372	285	329
Clinic visits				
Adult	914 (92.7)	344 (92.5)	257 (90.2)	313 (95.1)
Pediatric	72 (7.3)	28 (7.5)	28 (9.8)	16 (4.9)
Total, No.	986	372	285	329
Patients				
Adult	443 (94.1)	145 (92.9)	125 (94.7)	173 (94.5)
Pediatric	28 (5.9)	11 (7.1)	7 (5.3)	10 (5.5)
Total, No.	471	156	132	183
Sex				
Female	223 (47.3)	76 (48.7)	60 (45.5)	87 (47.5)
Male	248 (52.7)	80 (51.3)	72 (54.5)	96 (52.5)
Age, mean (SD), y	46.7 (19.1)	46.7 (18.4)	46.3 (19.8)	47.5 (19.3)
Race				
Asian	106 (22.5)	34 (21.8)	31 (23.5)	41 (22.4)
Black	12 (2.5)	2 (1.3)	5 (3.8)	5 (2.7)
White	289 (61.4)	98 (62.8)	75 (56.8)	116 (63.4)
Other^c^	64 (13.6)	22 (14.1)	21 (15.9)	21 (11.5)

^a^ Percentages represent the fraction of data within the combined data from all 3 time periods.

^b^ Percentages represent the fraction of data within the respective time period (eg, 2015-2016, 2016-2017, or 2018-2019).

^c^ American Indian, Native Hawaiian, Other Pacific Islander, other, unknown, and declined, according to data extracted from the electronic medical system.

**Figure.**
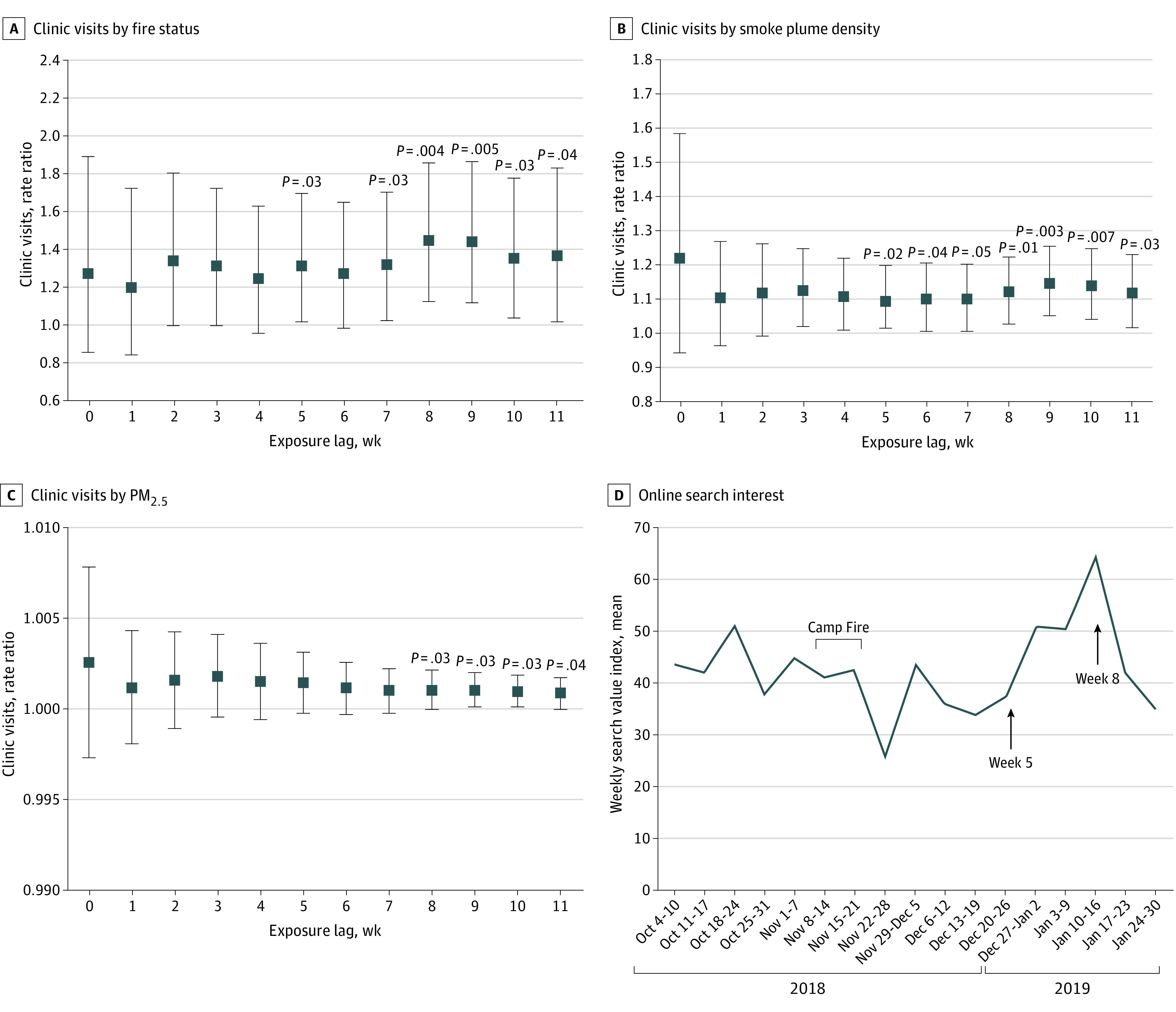
Clinic Visits and Online Search Interest for Psoriasis Before, During, and After the 2018 California Camp Fire A, Clinic visits by fire status. B, Clinic visits by smoke plume density. C, Clinic visits by particulate matter less than 2.5 μm in diameter (PM_2.5_). D, Online search interest for *psoriasis* in San Francisco in 2018-2019. Adjusted generalized Poisson regression results for weekly adult (≥18 years) clinic visits for psoriasis. Squares indicate the rate ratio, adjusted for weekly temperature, humidity, age, year, and overall patient volume at clinics. The error bars indicate 95% CIs. The lag considers cumulative exposure to air pollution. For PM_2.5_ and smoke plume density scores, the rate ratios are for a 1-unit and a 1-unit increase in the relevant scale, respectively. The Camp Fire increased air pollution in San Francisco for a 2-week period (November 8 to 21, 2018). Data on PM_2.5_ were obtained from ground air monitoring, and data on smoke plume density were obtained from the National Oceanic and Atmospheric Administration’s Hazard Mapping System Fire and Smoke Product.

## Discussion

Our findings demonstrate that air pollution from a wildfire was associated with modestly increased rates of clinic visits for psoriasis among adults starting 5 weeks after the fire and peaking at 8 to 9 weeks after the fire, supporting the association of air pollution with delayed psoriasis flares. This is consistent with the report of increased PM_2.5_ within 60 days before psoriasis flares.^1^ It also explains why the SVI for psoriasis did not increase during wildfires^5^; we report that online queries for psoriasis in San Francisco, which would likely increase as soon as skin symptoms appear, instead increased starting 5 weeks after the California Camp Fire. On a molecular level, air pollutants cause upregulation of several genes associated with inflammation and psoriasis, which could be associated with disease flare.^6^

Limitations of our study include the focus on only 1 wildfire affecting a city distant from its origin; thus, our findings likely underestimate the overall burden of skin disease associated with wildfires and communities experiencing greater smoke exposure. The low number of children in the study was likely associated with null findings for the pediatric population. Also, the minority racial and ethnic populations of San Francisco are proportionally underrepresented in our study, so our results may underrepresent the association of wildfire air pollution with psoriasis among those subgroups. Future studies should include other locations with wildfires, which will likely affect the medical services of many health care systems.^2^ Understanding environmental triggers is important for appropriately counseling patients regarding the risk of pollution-induced psoriasis flares as wildfires increase.

## Notes

Supplement 1.
eAppendix.

Supplement 2.
Data Sharing Statement

## References

[1] Bellinato F, Adami G, Vaienti S, . Association between short-term exposure to environmental air pollution and psoriasis flare. JAMA Dermatol. 2022;158(4):375-381. 35171203 10.1001/jamadermatol.2021.6019 PMC8851365

[2] United Nations Environment Programme. Spreading like wildfire: the rising threat of extraordinary landscape fires. 2022. Accessed May 20, 2022. https://wedocs.unep.org/bitstream/handle/20.500.11822/38372/wildfire_RRA.pdf

[3] Burke M, Driscoll A, Heft-Neal S, Xue J, Burney J, Wara M. The changing risk and burden of wildfire in the United States. Proc Natl Acad Sci U S A. 2021;118(2):e2011048118. doi:10.1073/pnas.2011048118 33431571 PMC7812759

[4] Fadadu RP, Grimes B, Jewell NP, . Association of wildfire air pollution and health care use for atopic dermatitis and itch. JAMA Dermatol. 2021;157(6):658-666. 33881450 10.1001/jamadermatol.2021.0179 PMC8060890

[5] Fadadu RP, Chen JY, Wei ML. Associations between wildfire air pollution and online search interest for skin diseases and symptoms. JAAD Int. 2022;8:128-130. 35875399 10.1016/j.jdin.2022.06.014 PMC9305359

[6] Cheng Z, Liang X, Liang S, Yin N, Faiola F. A human embryonic stem cell–based in vitro model revealed that ultrafine carbon particles may cause skin inflammation and psoriasis. J Environ Sci (China). 2020;87:194-204. 31791492 10.1016/j.jes.2019.06.016

