# Errors in Figure

**DOI:** 10.1001/jamanetworkopen.2023.1218

**Published:** 2023-02-15

In the Research Letter titled “Association of Wildfire Air Pollution With Clinic Visits for Psoriasis,”^1^ published January 13, 2023, the data plotted for lag 3 and lag 4 in panel B of the Figure showed a lower end of the 95% CI above 1 for both lag 3 and lag 4. However, the data plotted for the lower end of the 95% CI for each of these 2 lags should have been below 1. This article has been corrected.^1^

## References

[1] Fadadu RP, Green M, Grimes B, . Association of wildfire air pollution with clinic visits for psoriasis. JAMA Netw Open. 2023;6(1):e2251553. doi:10.1001/jamanetworkopen.2022.51553 36637821 PMC9857436

