# Supplementary material for: Association of Wildfire Air Pollution With Clinic Visits for Psoriasis
Source: JAMA Netw Open. 2023 Jan 13;6(1):e2251553. doi: 10.1001/jamanetworkopen.2022.51553 (PMC9857436; doi:10.1001/jamanetworkopen.2022.51553)
Supplement: Supplement 1. — eAppendix. [file jamanetwopen-e2251553-s001.pdf]

Supplementary Online Content

Fadadu RP, Green M, Grimes B, et al. Association of wildfire air pollution with clinic visits for psoriasis. *JAMA Netw Open.* 2023;6(1):e2251553. doi:10.1001/jamanetworkopen.2022.51553

eAppendix.

This supplementary material has been provided by the authors to give readers additional information about their work.

© 2023 Fadadu RP et al. *JAMA Network Open.*

eAppendix.

Data Sources:

Data on $PM_{2.5}$ were collected from the only Bay Area Air Quality Management District's ground-level air quality monitoring station in San Francisco, which provided daily 24-hour average $PM_{2.5}$ concentrations (μg /m^3). We collected daily smoke plume density scores from the National Oceanic and Atmospheric Administration (NOAA) Hazard Mapping System (HMS) for Fire and Smoke. Covariate data on the daily mean temperature and relative humidity were collected from the NOAA Local Climatological Data, and data on patient age, sex, and self-reported race/ethnicity were collected from the UCSF electronic health record system. Data on clinic visits were collected using codes from the *International Statistical Classification of Diseases and Related Health Problems, Tenth Revision* (*ICD-10*). The *ICD-10* codes used for psoriasis were L40.0, L40.1, L40.2, L40.3, L40.4, L40.8, L40.9.

Statistical Analysis:

We calculated the weekly sum of clinic visits for psoriasis (children age <18 and adults $\geq$ 18 years old) and the weekly average $PM_{2.5}$ concentration, smoke plume density score, temperature, relative humidity, and age of patients. We used a generalized Poisson regression model:

$$f(y_i, \mu_i, \alpha) = \left(\frac{\mu_i}{1 + \alpha\mu_i}\right)\frac{(1 + \alpha y_i)^{y_i - 1}}{y_i!} \cdot e^{\left[\frac{-\mu(1+\alpha y_i)}{1+\alpha\mu_i}\right]},$$

where $Y_i, (i = 1, 2, 3, \dots)$ is the number of appointments for psoriasis and α is a parameter which accounts for the variation in the number of appointments. The probability function of Y_i is given by: $y_i = 0, 1, 2, \dots$, and $\mu_i = \mu_i(x_i) = exp(x_i\beta)$, where x_i is a $(k - 1)$ dimensional vector of covariates including air pollution metric, average temperature, average humidity, average patient age, year, and holiday week. β is a k-dimensional vector of regression parameters.

© 2023 Fadadu RP et al. *JAMA Network Open.*

Each outcome was analyzed in models with a specific exposure as the primary predictor: fire status, smoke plume density score, or $PM_{2.5}$. Eleven 1-week cumulative exposure lags were constructed and analyzed independently in separate models. Covariates in the models included temperature, humidity, age, and time period (2015-2016, 2016-2017, or 2018-2019). Each model included an offset variable, which was the logarithm of the weekly total number of dermatology clinic visits for any skin health-related concern. Lastly, the models included a holiday week indicator variable, since the last full week of November 2015, 2016, and 2018 included the U.S. national Thanksgiving holiday, and clinics were partially closed during this time.

© 2023 Fadadu RP et al. *JAMA Network Open*.
