# Supplementary material for: Association of Wildfire Air Pollution With Clinic Visits for Psoriasis
Source: JAMA Netw Open. 2023 Jan 13;6(1):e2251553. doi: 10.1001/jamanetworkopen.2022.51553 (PMC9857436; doi:10.1001/jamanetworkopen.2022.51553)
Supplement: Supplement 2. — Data Sharing Statement [file jamanetwopen-e2251553-s002.pdf]

Data Sharing Statement

Fadadu. Association of Wildfire Air Pollution With Clinic Visits for Psoriasis. *JAMA Netw Open*. Published January 13, 2023. doi:10.1001/jamanetworkopen.2022.51553

Data
Data available: No

Additional Information
Explanation for why data not available: no patient consent to share data
